# The Challenge of Parenting Girls in Neighborhoods of Different Perceived Quality

**DOI:** 10.3390/soc4030414

**Published:** 2014-08-13

**Authors:** Lia Ahonen, Rolf Loeber, Alison Hipwell, Stephanie Stepp

**Affiliations:** Life History Program, Department of Psychiatry, University of Pittsburgh, 201 N Craig Street, Suite 408, Pittsburgh, PA 15218, USA

**Keywords:** girls, parental engagement, neighborhoods, delinquency

## Abstract

It is well-known that disadvantaged neighborhoods, as officially identified through census data, harbor higher numbers of delinquent individuals than advantaged neighborhoods. What is much less known is whether parents’ perception of the neighborhood problems predicts low parental engagement with their girls and, ultimately, how this is related to girls’ delinquency, including violence. This paper elucidates these issues by examining data from the Pittsburgh Girls Study, including parent-report of neighborhood problems and level of parental engagement and official records and girl-reported delinquency at ages 15, 16, and 17. Results showed higher stability over time for neighborhood problems and parental engagement than girls’ delinquency. Parents’ perception of their neighborhood affected the extent to which parents engaged in their girls’ lives, but low parental engagement did not predict girls being charged for offending at age 15, 16 or 17. These results were largely replicated for girls’ self-reported delinquency with the exception that low parental engagement at age 16 was predictive of the frequency of girls’ self-reported delinquency at age 17 as well. The results, because of their implications for screening and early interventions, are relevant to policy makers as well as practitioners.

## 1. Introduction

Parenting practices and community factors are two popular explanations of juvenile delinquency, including violence, however not without controversy. A wide array of research has focused on parental styles [1,2] separately from community factors [3,4] such as socioeconomic status, racial distribution and demographics. However, fewer studies have focused on how neighborhood qualities affect parents’ parenting strategies [5]. Interestingly, studies do not show unanimous results when it comes to how neighborhood problems affect girls’ compared to boys’ delinquency [6]. Even rarer are studies of the relationships between parents’ *perception* of neighborhood problems [7], neighborhood quality [8], parents’ engagement with their child, such as supervision and involvement, and girls’ juvenile delinquency, and how stable these factors are over time.

It is often assumed that disadvantaged neighborhoods and parenting practices predict delinquency in the offspring, but little is still known about whether this is true for girls, and if the relationship changes with age. It is also well known that disadvantaged compared to advantaged neighborhoods harbor more risk factors for delinquency, and that both juvenile and parents’ problem behaviors such as delinquency, drug use, low employment rates, are overrepresented in these areas [9]. According to one train of thought, parents in disadvantaged neighborhoods are likely to be exposed to numerous risk factors that negatively affect the quality of the parenting of their offspring. Another view would be that parents living in disadvantaged neighborhoods often are aware of the dangers for their children in those settings and practice strict parenting to protect their children. To our knowledge, there are few studies investigating how the level of *perceived neighborhood problems* is related to parental engagement with the girl, and the extent to which this parenting predicts girls’ delinquency. Being a good parent is challenging, and even more so in disadvantaged neighborhoods.

## 2. Previous Research and Current Focus

Different types of poor parenting are well-documented as risk factors for delinquency in the offspring [1,10,11]. In a meta-analysis of the relationship between parenting and delinquency Hoeve *et al.* [12] found that the strongest associations with delinquency were parental monitoring (supervision), psychological control and lack of support (involvement). In the current study, we use the term parental engagement to describe the process that constitutes both supervision and involvement.

Parenting does not exist in a vacuum, but often takes place in response to neighborhood characteristics [13] and together with the home and the school situation constitutes the child’s overall learning arena. This is where future behavior and moral standards are shaped and tested in environments that may vary in terms of safety and degree of disadvantage [14]. Parents play a central role in this complicated system, and are often considered to have a causal impact on their children’s behavior. We now know that it is not just sufficient to have caregivers *present* in the children’s life, but that the *quality* of the parent-child relationship is what counts [15].

Parents also deal with societal expectations on child practices that are often related to gender specific developmental factors. Altogether, this has inspired researchers for almost a century to suggest that parents socialize their female and male offspring differently, according to expected gender roles [16,17]. Thus, girls tend to be more strictly supervised than males during childhood [18,19], spend more time playing inside the family home rather than in the neighborhood outside of the parent’s control [20], and therefore have less opportunity to interact with deviant peers and engage in co-offending. We also know that there are differences in the expression of delinquency in females compared to males; for example, female violence is often more directed towards family members than strangers [21]. Girls compared to boys may need to adjust to stricter supervision by parents and a more closely tied social structure. Further, we need to know how parents’ perception of neighborhood problems may affect the supervision of and involvement with their girls, and how that may affect girls’ well-being, including their delinquency.

### 2.1. Supervision and Involvement

Compared to parents’ involvement, supervision is the more instrumental aspect of the two concepts (closely related to socialization and compliance [14]) and consists of parents setting rules for the child to live by. Examples of such rules are which friends to play with, when to return home, what way to walk to school, how to talk to adults, how to behave in school and many other daily routines. Involvement is a more complicated process and is known under many different names and includes parent’s rejection of their child and positive parenting [1]. Involvement compared to supervision often has more of an emotional component. In some studies, including the current study, involvement is also a measure of how much time parents spend with the child. Parents who are highly involved in their child’s life tend to influence their child’s norms and values to a high degree, whether positive or negative [15]. More specifically, the quality of the relationship between parent and daughter is crucial for how much of the parents’ norms and values are passed on to children. If the relationship is strong, and the parents are pro-social, parents are more likely to pass on positive values. If, on the other hand, the parent/s are anti-social they are more likely to convey negative norms and values to their daughter.

### 2.2. The Present Study

We examined how parental engagement was related to perception of neighborhood problems and how each factor might be related to girls’ delinquency including violence. The prevalence of violence in girls was much lower than in male samples and therefore we have not separated violent and non-violent behaviors in this study. Using longitudinal data from when the girls were 15, 16 and 17 years old we employed structural equation modeling (SEM) to examine the temporal order of neighborhood problems, parental engagement (supervision and involvement) and their girl’s delinquency. We chose these ages because of several reasons. First, girl’s delinquency tends to peak earlier than boys, and therefore we do not include later ages [22]. Second, mid adolescence is the period where juveniles detach most from their parents [14], and therefore we expect to find more significant associations at younger than older ages. Three, juvenile delinquency records cease after age 17 and parents were not interviewed once girls turned 18. The study addresses the following questions:

1. To what extent are parents’ perception of neighborhood problems, parental engagement and girls’ delinquency stable over time?
2. Does parents’ perception of neighborhood problems predict parental engagement in the girl’s life?
3. Does parental engagement mediate the relationship between parents’ perception of neighborhood problems and their girl’s delinquency?
4. To what extent do the results apply equally to official records of charges for offending and self-reported delinquency?

## 3. Methods: Sample and Procedure

The Pittsburgh Girls Study is a longitudinal panel study that started in 2000, which follows up girls from the city of Pittsburgh. The total sample consists of four cohorts (initial ages 5, 6, 7, 8). Since it is well known that the prevalence of female delinquency is much lower compared to that of males, oversampling was performed in the most disadvantaged neighborhoods (100% in disadvantaged neighborhoods, 50% in advantaged neighborhoods, according to US Census data). In total 2451 girls participated in the study (approximately 600 in each cohort [23]). Since the vast majority (93%) of the caregiver respondents was the girls’ biological mother, we refer to all caregiver respondents as “parent” in the text. The study had a high participation rate of parents and girls (ranging from 85.4% to 85.6% between ages 15 and 17). Valid data on delinquency charges ranged between 91%–93%, due to missing cases that moved out of the county or were deceased.

For this study, we used data from all four cohorts at ages 15, 16 and 17. Over these years we used information from parents about their perception of neighborhood problems, and parents’ self-reports of their engagement with their girl. Further we used official records to investigate whether the girls had been charged with any type of serious offenses (see measurement section for exclusion criteria) at any of the time points together with girls’ self-report on delinquency.

There are multiple rationales for choosing this particular age range. One was to achieve consistency among measurements; more specifically all of the instruments were used at these ages. Another reason was that we believe that neighborhood is more important at those ages than at earlier ages, due to the fact that the girls would interact more independently with neighborhood factors. This in turn might have an influence on the parent’s perception of the neighborhood. On the other end of the scale, we did not go beyond age 17, due to lack of completed collection of adult criminal records (Age 18 and later). Of the 2451 girls, 41.2% were Caucasian white, 52.9% were African American and 5.9% were reported as other race of origin. When the girls were 15 years old, 37.9% of parents were white, 45.7% were African American, and 16.3% reported other racial background.

## 4. Measurements

### 4.1. Perceived Neighborhood Problems

Neighborhood problems were reported by parents when the girls were 15, 16 and 17 years respectively, on the basis of the Your Neighborhood questionnaire [24]. This scale consisted of 17 items (age 15: *n* = 2129, α = 0.96; age 16: *n* = 2087, α = 0.95; age 17, *n* = 2042, α = 0.95). The parents were asked to rate how much of a problem a number of issues were in their neighborhood. Examples of rated items were: unemployment, groups not getting along with each other, vandalism, prostitution, sexual assaults or rapes, burglaries, assaults, delinquent gangs, and drug use. The answer categories were: not a problem, somewhat a problem, and big problem.

### 4.2. Parental Engagement

The construct of parental engagement consisted of the two dimensions called supervision and involvement. Parental supervision and involvement were measured at ages, 15, 16 and 17. Both aspects of supervision and involvement were measured by means of the Supervision Involvement Scale (SIS) developed by the Pittsburgh Youth Study research group [24]. Parents’ self-reported engagement was based on 14 questions (age 15, *n* = 1912, α = 0.81; age 16, *n* = 1923, α = 0.79; age 17, *n* = 1821, α = 80). Examples of supervision questions were: When was the last time you discussed with your daughter [girl] her plans for the coming day? Do you know who your daughter’s companions are when she is not home? Examples of involvement questions are: Do you find time to listen to your daughter when she wants to talk to you? How often do you have a friendly chat with your daughter? The response alternatives were for some items a time scale ranging from every day, to once a month, or more seldom, and for some items almost never, sometimes, or often.

### 4.3. Girl’s Delinquency

#### 4.3.1. Official Records

Delinquency was measured through official charges for offending at ages 15, 16 and 17. Any charge yields a single score. Included were all offenses (violent and non-violent) that would yield a felony as an adult (criminal activity). Examples of records were aggravated assault, carjacking, robbery, weapon possession, burglary, sale or delivery of drugs, vandalism, disorderly conduct, retail theft, dealing with stolen goods. Examples of excluded items were records of status offenses, such as running away from home, under-age drinking, or minor traffic violations.

#### 4.3.2. Self-Reported Delinquency

Delinquency was also measured through yearly self-reports at ages 15, 16 and 17 using the (SRD) developed by the Pittsburgh Youth Study group [24]. We created a scale of self-reported delinquency for ages 15, 16, and 17. Examples of items were: How many times did you carry a concealed weapon during the last twelve months? How many times did you destroy others property during the last 12 months? How many times did you sell marijuana during the last 12 months? How many times were you involved in assaulting someone during the last 12 months? The response alternatives ranged from zero to n (no upper limit). The self-reported delinquency was then divided in the frequency of delinquent acts up until 14 times then a cut off was created and named “15 times or more”. The reason of doing this is to avoid extreme outliers, without deleting cases.

## 5. Analytic Strategy

The PGS is a panel study with repeated measures, which makes it suitable to test a longitudinal mediation model. Female delinquency has a relatively low prevalence and its distribution is highly skewed, with the majority of individuals endorsing a zero count for delinquency. Under these circumstances, the traditional OLS regression would likely lead to a violation in the assumption of normality in the distribution of residuals, and is therefore not suitable [25,26]. With a large portion of zero’s and the remaining observations following a count distribution, it is advisable to use a zero-inflated Poisson (ZIP) model [25]. ZIP models can be estimated in a regression or path-modeling framework, and they account for the large number of zero counts (e.g., no delinquency) compared to number of delinquent activities (e.g., delinquency severity or frequency). This is accomplished by using a special form of a mixture model, which divides the sample in to those who are in the zero class (*i.e.*, no delinquency) and those that have the potential to exhibit some degree of the behavior of interest (*i.e.*, a frequency count of the delinquent class). Therefore, we estimated two sets of regression parameters, first, logistic parameters are used for variables that are included in the models to differentiate between classes (*i.e.*, non-delinquent *vs.* some degree of delinquency), and second, Poisson regression parameters for predictors of severity of delinquency given membership in the delinquent class. Importantly, there is no assumption that the predictors and patterns of significance across sets of parameters need be the same. In other words, this type of model yields two sets of outcome variables: (1) presence *vs.* absence of delinquency (charges or self-reports); and (2) if present to what degree of severity (number of charges or self-reported delinquent acts). Each of these can then be predicted by the same or different sets of variables.

Path models were used to test the impact of neighborhood problems, as reported by parents at age 15 and 16, on parental engagement at age 16 and 17, and neighborhood problems and parental engagement at age 15 and 16 as mediating predictors of the girl’s later contacts with the justice system at age 16 and 17 or their self-reported delinquency, respectively. The analyses proceeded in three steps, first testing the impact of neighborhood problems on delinquency, second testing the effect on perception of neighborhood problems on parental engagement, and finally parental engagement on both presence and severity of delinquency. More specifically, three regression models were estimated in Mplus [27]. We first tested a model where delinquency was regressed on neighborhood problems. Second, we regressed delinquency on parental engagement. In the two final models, delinquency (official charges and self-reported delinquency respectively) was regressed on parental engagement, and parents’ report of neighborhood problems (see Figure 2). Race was included as a covariate.

Table 1 shows the intercorrelations between the study variables. Most of the correlations are in the low range, the exceptions are perception of neighborhood problems and parental engagement (Neighborhood ranging from *r* 0.62–0.71; Parental engagement *r* 0.52–0.60).

## 6. Results

### 6.1. Prevalence of Delinquency

The cumulative prevalence of officially violent and nonviolent (including substance use related charges) charges in girls over three waves/panels, amounted to 13.4%. Self-reported delinquency over three age categories (ages 15, 16 and 17) show that 46.4% reported at least one delinquent act and 12.7% reported 5 delinquent acts or more during the previous 12 months. Table 2 shows the prevalence of different categories of delinquency both from official records (charges) and self-reported delinquency.

### 6.2. Continuity over Time

**Figure 1:**
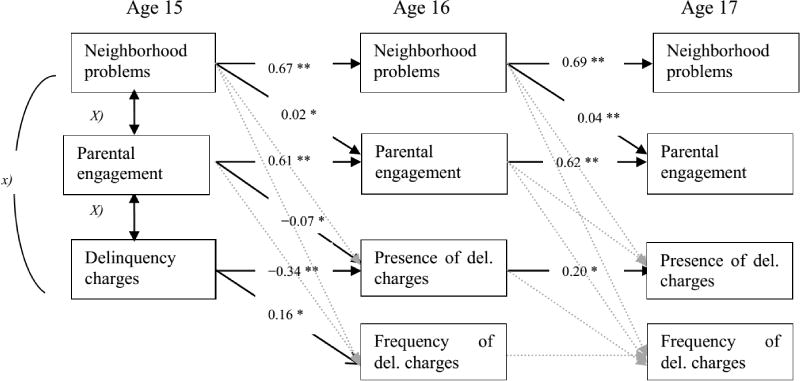
Estimators from the final path way model using delinquency charges. Note: ^*^p ≤ 0.05; ^**^p ≤ 0.001; *Correlations between key variables are reported in* Table 1. Tested but non-significant paths are illustrated with light grey dotted lines.

**Figure 2:**
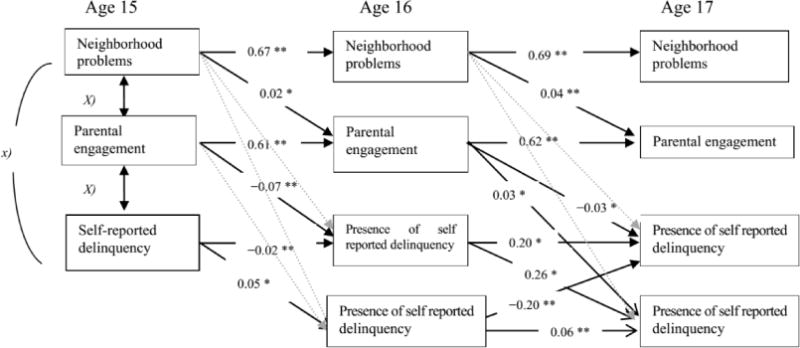
Estimators from the final path way model using self-reported delinquency. Note: ^*^p < 0.05; ^**^p < 0.001; Correlations between variables are reported in Table 1. Tested but non-significant paths are illustrated with light grey dotted lines.

Figures 1 and 2 show the models tested for delinquency charges and self-reported delinquency, respectively. The first question we raised was: To what extent are parents’ perceptions of neighborhood problems, parental engagement, and girls’ delinquency stable over time? Figures 1 and 2 indicate higher stability for parental perception of neighborhood problems and parental engagement than girl’s delinquency. Whereas delinquency charges and self-reports of delinquency at age 15 negatively predicted the same outcomes at age 16, respectively (however, the regression estimates were modest to very small; −34, *p* < 0.001; −0.02, *p* < 0.001), delinquency charges and self-reported delinquency at age 16 positively predicted the same outcomes at age 17, respectively (see Figures 1 and 2). Thus, the results only partially confirmed our expectations.

The second question we addressed was: Does parents’ perception of neighborhood problems predict parental engagement in the girl’s life? We found that the parent’s perception of the neighborhood problems predicted their level of engagement in the girls’ life from age 15 to 16 and from age 16 to 17. However, the magnitude of the regression estimates were significant but small (0.02, *p* < 0.001; 0.04, *p* < 0.001, respectively) when other factors were controlled in the models (see Figures 1 and 2).

The next question addressed was whether parental engagement mediated the association between parents’ perception of neighborhood problems and girl’s delinquency. Figure 1 shows that the contemporaneous association between parents’ perception of neighborhood problems and parental engagement at age 15 negatively predicted the presence of girls’ charges for offending, but were not significantly related to charges at age 16. Turning to self-reported delinquency, the above results for age 15 were replicated and indicate in that high parental engagement predicted self-reported delinquency in the girls better than official charges at that age (Figure 2). High engagement by the parent was associated with high self-reported delinquency in the girls. This is contradictory to what was we expected, and indicate that supervision and involvement in terms of control and spending time together can have a contra productive influence on children’s adjustment. Figure 2 also tests the mediation model between parents’ perception of neighborhood at age 15, parents’ engagement at age 16, and the girls’ self-reported offending at age 17. The results partially support the mediation model, in that parental engagement at age 16 positively predicted frequency of girls’ self-reported delinquency at age 17. However, parental engagement at age 16 was negatively predictive of the presence of self-reported delinquency. Thus, low engagement by the parent was related to higher frequency of self-reported offending over time.

Finally we addressed the question: To what extent do the results apply equally to girls’ self-reported delinquency and girls’ official records of charges for offending? We found in our analysis notable similarities between the two models (Figures 1 and 2) using delinquency charges and self-reported delinquency. The models have 10 statistically significant paths in common, and 5 unique paths in the model using self-reported delinquency as outcome. Thus, most of the results applying to girls’ charges for offending also applied to girls’ self-reported delinquency as an outcome.

## 7. Discussion

The stability over time for neighborhood problems and parental engagement was moderate to high, in contrast to lower continuity of delinquency variables. Official charges only represent those who get caught and are registered in the juvenile justice system, and therefore we used self-reports as an additional source of information. Delinquency, especially for females, tended to fluctuate during adolescence and girls’ delinquency tended to peak earlier for girls than for boys [14]. In this study we found that of the ages studied, delinquency was highest at age 15 and decreased subsequently, especially for self-reported offending (which conforms to the age-crime curve [14]). We also found that the stability of delinquency was higher between ages 15 and 16 than between ages 16 and 17 (this applied to both girls’ charges for offending and their self-reported delinquency), which agrees with the theme of decreasing prevalence of offending with age. The self-reports show interesting results where presence of delinquency at age 16 predicts escalation one year later, but a high frequency of delinquent activities at age 16 did not predict presence of delinquency at age 17. This is comparable to previous studies showing that girls have shorter and often intermittent delinquency careers than boys do [16,28].

## 8. Conclusions

In the introduction we contrasted two ideas about parenting in different types of neighborhoods. One is that parenting is better in disadvantaged neighborhoods, because parents are aware of the dangers for their children. A contrasting idea is that parenting is worse in disadvantaged compared to advantaged neighborhoods because of the higher concentration of multiple-problem families, and risk factors, and this is more impairing for parenting strategies including parental engagement.

What is a bad neighborhood? Already in the early decades of the twentieth century, criminologists suggested that the closest environment and neighborhood were crucial for criminal or pro social behavior [3,4]. People in disadvantaged neighborhoods are not different from other people, when population turnover is high; the problems tend to stay within the neighborhood and its residents feel less safe. It is not low average income, uneven distribution of race, or fewer resources to schools alone, but all of these factors taken together that lead to a less livable neighborhood. Albert Reiss wrote [23] (p. 2) that “Dangerous places sometimes identify themselves by the visible signs of crime environments such as broken windows, graffiti, vandalized property and drawn iron gates. Still, the common way to think about safe and dangerous communities is to think of them as aggregations of law-abiding and criminal persons”. The current paper does not focus on bad neighborhoods as defined by for example Census, but people’s subjective perceptions. Therefore we did not test for possible differences between low income areas and other areas.

Often the role of the community in shaping citizen morality is highly under-estimated [29,30], and is seldom discussed in relation to parenting and the inevitable interaction with the community. In the current study we found stronger support for parental involvement mediating the impact of neighborhood problems on girls’ self-reported delinquency than on girls’ charges for offending. The results suggest that between ages 15 and 16, parents’ engagement can be counterproductive in that it was associated with higher than lower presence of delinquency (charges and self-reports). However, the magnitude of the coefficients was small when effects were tested within the models. On the other hand, high parental engagement was predictive of lower frequency of girls’ self-reported offending at age 17. It may well be that different aspects of parental engagement, such as supervision and involvement are differently related to girls’ offending later.

One of the key questions in this study was whether overall the results would replicate when using official charges and self-reported delinquency, despite the prominent difference in prevalence and correspondence between the two. We found that over all, the model replicated well.

### 8.1. Limitations and Directions for Future Research

We used data only from ages 15, 16 and 17, and future studies should expand this age span to relate the results better to the age-crime curve. In addition, we used only parents report on neighborhood problems and their parental engagement; shared method variance may account for some of the results. Further, future studies should investigate further whether parents’ perceptions of neighborhood problems correspond with for example Census’ ratings of neighborhood problems. In addition, studies should investigate whether parents’ own characteristics (anxiety, depression and other problems) affect how they rate the neighborhood, and thus how this influence their level of parental engagement. We also measured only the overall risk and not the positive aspects of support in the neighborhood, which could be an important part of overall perception of neighborhood problems. Further, the results indicate that there can be differences in the way parents perceive their own supervision of their girls, and the actual level of supervision, as well as the inevitable interchange between parent and child where the girl also affect the parents behavior in a transactional model [31]. This argument is supported by Stattin and Kerr [32], who stated that parental knowledge of their child’s behavior does not only originate from instrumental supervision, but from voluntarily disclosure from the child in a reciprocal process that, can only be facilitated through a high quality engaged relationship. Thus, future studies should focus on investigating the mechanisms behind supervision and involvement and the voluntarily shared information that girls provide their parents. The key issue is how to increase disclosure and communication between parent and child. We also recommend that future studies test the two competing hypothesis: parents who perceive their neighborhood as less positive supervise their children more strictly, or they show less parental engagement (supervision and involvement combined) due to lack of resources (personal, financial and societal) and support. In addition, future studies should investigate whether parental engagement is differently associated with delinquency in different types of neighborhoods.

### 8.2. Implications

These results suggest that it is important to prioritize communication between parent and child in intervention initiatives, such as family and parent training programs. Parents need to be aware of the cognitive and developmental discrepancies between themselves and their children, and that adolescent girls not always share their thoughts with their parents even if the relationship is positive. This often leads to parents assuming that their daughter is hiding things from them, and that they need to increase control and supervision. In a normal development, adolescent girls strive to become more autonomous from their parents during mid to late adolescence, and parents also need to be aware that this is a part of the natural developmental process toward autonomy. Therefore, instead of demanding complete disclosure from the girl that instead could lead to conflict, intervention programs should focus on training parents to recognize when to apply control and when not.

The results also demonstrate the divergence of some of the findings on official charges for offending and self-reported delinquency. Intervention programs to aid parents should incorporate the large differences between official records and self-reported delinquency because many delinquent girls stay undetected which can become an obstacle for screening and offering interventions.

Interventions focusing family and parental training should include an awareness of age effects and girls presumably higher level of autonomy compared to boys in mid and late adolescence [33]. Parents need to deal with their own concerns about the neighborhood they live in and support but not hinder the girl to interact with the close environment. Thus, parents’ perception of the neighborhood they live in should be acknowledged in parenting training programs and other prevention strategies, such as programs distributed through public schools. Policy makers should encourage community interventions in not only the worst disadvantaged neighborhoods, but on a universal prevention level, through general programs distributed through public schools [34]. These programs should include both parent training and their interaction with neighborhood factors.

In a recent report, Huizinga *et al.* [28] showed that girls are much more difficult to detect in the context of delinquency, compared to boys, due to lower prevalence, differing developmental patterns and more “scattered” initiation processes (age, type of delinquent acts) into delinquency [28]. The current study lends support to these assumptions, since there is most likely an age effect where female delinquency peaks earlier and the girls are less influenced by their parents. Parental engagement needs to adjust according to girls’ rapid development of autonomy, in order to be influential over time and in the later years of adolescence and into early adulthood.

## Figures and Tables

**Table 1:** Intercorrelations between key variables included in the two final models.

	N15	N16	N17	P15	P16	P17	D15	D16	D17	DS15	DS16	DS17
N15		0.71**	0.62**	0.10**	0.11**	0.10**	0.03	0.06**	0.06**	0.03	0.01	0.07**
N16			0.69**	0.10**	0.12**	0.14**	0.03	0.09**	0.03	0.05*	0.05*	0.06*
N17				0.09**	0.10**	0.11**	0.018	0.09**	0.03	0.04	0.04	0.05*
P15					0.60**	0.52**	0.05*	0.09**	0.05*	0.03	0.05*	0.06*
P16						0.60**	0.06**	0.14**	0.05*	0.01	0.06**	0.07**
P17							0.04	0.11**	0.03	0.07**	0.07**	0.08**
D15								0.14**	0.09**	0.12**	0.06*	0.08*
D16									0.07**	−0.01	0.01	0.05*
D17										−0.01	0.05	0.06
DS15											0.33**	0.18**
DS16												0.28**
Girls Race 15	0.20**	0.20**	0.16**	0.09**	0.10**	0.09**	0.10**	0.12**	0.12**	0.05*	0.032	0.03
CGR15	0.20**	0.18**	0.17**	0.08**	0.08**	0.09**	0.08**	0.08**	0.05**	0.06*	0.05*	0.05*

Note: 15, 16 and 17 refers to the girls’ age; N = Neighborhood perception; P = Parental engagement; D = delinquency charges; SD= frequency of self-reported delinquency and CGR parents’ race;

* *p* < 0.05;

** *p* < 0.001.

**Table 2:** Delinquency prevalence rates at each age.

Official charges	Age 15 (*n* = 2451)	Age 16 (*n* = 2451)	Age 17 (*n* = 2451)
Total count (Any)	5.1%	5.6%	4.4%
-violent	2.7%	2.9%	2.5%
-non-violent	2.4%	2.7%	1.9%
Self-reported delinquency	(*n* = 2115)	(*n* = 2073)	(*n* = 2055)
Total count (Any)*	19.8%	17.2%	13.9%
-violent	11.5%	8.2%	6.4%
-non-violent	12.8%	12.7%	10.1%

* The percentages presented here add up to more or less than 100% because some girls reported both violent and non-violent offenses.
